# Effective methods to enhance medical students’ cardioversion and transcutaneous cardiac pacing skills retention - a prospective controlled study

**DOI:** 10.1186/s12909-022-03495-4

**Published:** 2022-06-01

**Authors:** Christian Kowalski, Anne-Laure Boulesteix, Sigrid Harendza

**Affiliations:** 1 Department of Anesthesiology, Ludwig-Maximilians-University, Munich, Germany; 2 Department of Medical Information Processing, Biometry and Epidemiology (IBE), Ludwig-Maximilians-University, Munich, Germany; 3 Department of Internal Medicine, University Medical, Center Hamburg-Eppendorf, Hamburg, Germany

**Keywords:** Objective structured clinical examination (OSCE), Performance, Skill retention, Skills training, Standard operating procedure (SOP), Synchronized cardioversion, Transcutaneous pacing

## Abstract

**Background:**

Guideline-based therapy of cardiac arrhythmias is important for many physicians from the beginning of their training. Practical training of the required skills to treat cardiac arrhythmias is useful for acquiring these skills but does not seem sufficient for skill retention. The aim of this study was to compare different retention methods for skills required to treat cardiac arrhythmias with respect to the performance of these skills in an assessment.

**Methods:**

Seventy-one final-year medical students participated in a newly designed workshop to train synchronized cardioversion (SC) and transcutaneous cardiac pacing (TCP) skills in 2020. All participants completed an objective structured clinical examination (OSCE 1) one week after the training. Afterwards, the participants were stratified and randomized into three groups. Nine weeks later, one group received a standard operating procedure (SOP) for the skills, one group participated in a second workshop (SW), and one group received no further intervention (control). Ten weeks after the first training, all groups participated in OSCE 2.

**Results:**

The average score of all students in OSCE 1 was 15.6 ± 0.8 points with no significant differences between the three groups. Students in the control group reached a significantly (*p* < 0.001) lower score in OSCE 2 (-2.0 points, CI: [-2.9;-1.1]) than in OSCE 1. Students in the SOP-group achieved on average the same result in OSCE 2 as in OSCE 1 (0 points, CI: [-0.63;+0.63]). Students who completed a second skills training (SW-group) scored not significantly higher in OSCE 2 compared to OSCE 1 (+0.4 points, CI: [-0.29;+1.12]). The OSCE 2 scores in groups SOP and SW were neither significantly different nor statistically equivalent.

**Conclusions:**

Partial loss of SC and TCP skills acquired in a workshop can be prevented after 10 weeks by reading an SOP as well as by a second workshop one week before the second assessment. Refreshing practical skills with an SOP could provide an effective and inexpensive method for skills retention compared to repeating a training. Further studies need to show whether this effect also exists for other skills and how frequently an SOP should be re-read for appropriate long-term retention of complex skills.

## Background

Some skills medical students acquire during undergraduate medical education are not frequently needed for patient management, even during postgraduate education [1]. Nevertheless, it is important to correctly perform such procedures, especially in case of an emergency. For cardiac arrhythmias, electric therapy, i.e., synchronized cardioversion (SC) and transcutaneous cardiac pacing (TCP), are challenging procedures for young physicians [2]. Unfortunately, most undergraduate students cannot learn SC and TCP during bedside teaching because unstable arrhythmias are rare emergency situations and must be treated immediately [3, 4]. Therefore, both therapeutic interventions need to be practiced in workshops using manikins and real defibrillators, because they require delicate handling of intravenously placed equipment and defibrillator/pacemaker, which cannot be practiced with real patients in an emergency situation [5]. Electric therapy of arrhythmias is often used in the emergency room and in the operating room, where many young residents start their careers. Therefore, acquiring these skills during undergraduate education seems to be a desirable goal. If these skills are performed incorrectly, e.g. if cardioversion is not synchronized, it can lead to deadly ventricular fibrillation in the patient or, if the defibrillator is triggered carelessly, a team member could receive an electric shock [6]. Correctly performed TCP increases a patient’s blood pressure and heart rate and thereby prevents possible brain damage [3, 6].

Acquired skills deteriorate quickly if routine training cannot be offered. Performance in neonatal resuscitation, for instance, reached only about 50% of the baseline 12 weeks after the initial training [7]. Deterioration in acquired skills was also observed in adults performing basic life support (BLS) [8, 9] or after a simulator training for transvenous pacing [10]. Even simple skills, e.g., placing a periphery venous catheter, showed significant and relevant reduction in performance, if students were not able to practice it frequently [11]. However, continuously repeated training in BLS enhanced the participants’ performance with the strongest effect when training took place monthly [12]. Several other methods were tested to increase the retention of skills because repeated training is expensive and requires personnel. For instance, non-medical students showed better performance in BLS 3 months after the initial training if they were reminded of the skill by a short video on their smartphones [13]. When nursing students were allowed to watch their own videos recorded during a BLS workshop 6 months earlier, they also showed better performance of this skill [14]. Feedback given during a cardiopulmonary resuscitation (CPR) workshop augmented medical students’ CPR performance and this effect was detectable even 12 months after the initial training [15]. In undergraduate dental education, repeated testing produced better skills retention than repeated practice [16].

In hospitals, many procedures are regulated by so called standard operating procedures (SOPs), short, but detailed and concisely written instructions that describe a specific, relevant activity to guide its uniform performance [17]. It has been found that the clinical behavior of emergency physicians changed positively after the introduction of an SOP [18]. It has also been shown that the introduction of an SOP regarding the treatment of patients with acute coronary syndrome had a positive effect on physicians’ adherence to guideline-related drug therapy [19]. Establishing an SOP for a cardio-oncology echocardiography team improved precision of measurements to detect cancer therapy-related cardiac dysfunction [20]. Therefore, we wondered whether reading an SOP could be used as learning tool for skills retention of SC and TCP in undergraduate medical education.

In this study, we established a workshop to teach medical interns two infrequently needed but important skills, SC and TCP. A few weeks later, some interns received reading material about these skills and other interns practiced these skills in a second workshop. Ten weeks after the first workshop, these skills were tested in an OSCE. Our research questions were: 1) Does performance of these skills deteriorate 10 weeks after a workshop? 2) Does reading a standard operating procedure (SOP) about these skills or participating in a second workshop one week prior to the OSCE lead to better retention of these skills?

## Methods

### Study design and participants

We carried out a prospective, stratified, controlled study with 71 interns (i.e. final-year undergraduate medical students in year 6 of the studies) in anesthesiology to investigate the influence of different interventions on the retention of specific, infrequently needed skills. Five participants dropped out for various reasons (one because of illness, two due to travel restrictions or quarantine during the corona pandemic, and two without reason). In total, 66 students finished the study, and their data could be included in the analysis. The study was conducted in 2020 – from January until October – and was performed in accordance with the Declaration of Helsinki and approved by the Ethics Committee of the Ludwig-Maximilians-University, Munich. It confirmed the innocuousness of the study with consented, anonymized, and voluntary participation (19–802). All participants provided informed written consent for participation in this study.

### Study procedure and setting

**Fig. 1:**
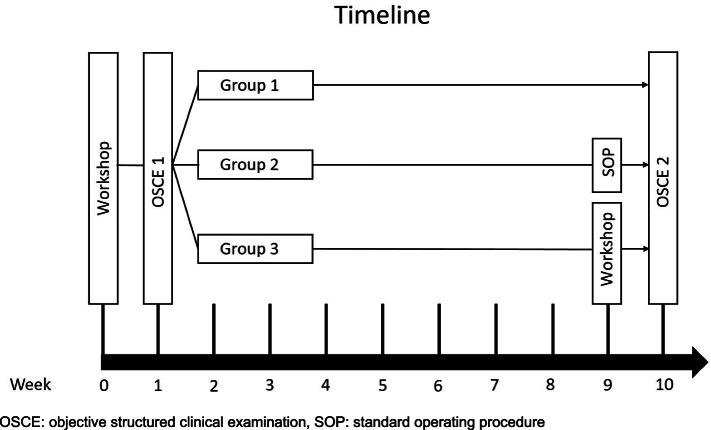
Timeline of the study

We instructed the 66 participants in a 90-minute workshop to treat “patients” with unstable tachycardia with synchronized cardioversion (SC) or unstable bradycardia with transcutaneous cardiac pacing (TCP). The participants were divided into small groups of three to six students (Fig. 1). They practiced the diagnosis and the electric therapy of different unstable arrhythmias on a simulation manikin (Resusci Anne Simulator, Laerdal Medical, Stavanger, Norway). The cost of one workshop for six participants including administration and material is approximately 400 euros. During the structured workshop which follows a standardized concept of instruction and practice including specific group sizes and equipment, all participants were given the opportunity to treat one “patient” with tachycardia und one “patient” with bradycardia. Questions were allowed at any time point. All interns were tutored by the same instructor. One week after the workshop all participants were examined in a first objective structured clinical examination (OSCE 1). They had to demonstrate SC and to perform a TCP, which were rated with a checklist (0–17 points for both tasks together). All students were tested by the same rater (OSCE 1). No feedback was given to the OSCE participants for standardization and to prevent possible bias in further assessments. For the second part of the study the students were split into three groups: one group received a standard operating procedure (SOP) for the skills, one group participated in a second workshop (SW), and one group received no further intervention (control).

### Group assignment

To reach an even distribution of male and female students as well as a similar mean OSCE 1 score in the three groups for the second part of our study, we used the following approach. According to their individual OSCE 1 scores, the interns were allocated in principle randomly to one of the three groups. The first half of the participants were assigned by simple randomization and the latter half were assigned to groups by hand such that the number of female participants and average OSCE 1 score were approximately the same across all three groups (control group: 15.6 ± 0.9 points, SOP-group: 15.6 ± 0.7, and SW-group: 15.7 ± 0.9). The control group (18 interns) received no further intervention, the SOP-group (24 interns) was asked to read a standard operating procedure (SOP) for SC and TCP, and the SW-group (24 students) participated in a second workshop. The interns of the SOP-group were sent a standard operating procedure (SOP) with detailed written instructions, four pages including pictures and stepwise approach on how to perform SC and TCP, 9 weeks after the workshop via e-mail. They were asked to prepare with the SOP for OSCE 2 one week later. The participants of the SW-group were invited to participate in a second workshop 9 weeks after the first training. The second workshop was identical to the one before. Ten weeks after the initial workshop, all interns participated in OSCE 2, where they had to demonstrate an SC and to perform a TCP again. OSCE 2 was identical to OSCE 1 (the same tasks, the same arrhythmias). The three raters of the second OSCE were “blinded” with respect to the participants’ group. After OSCE 2, individual feedback on their performance was given to all participants.

### Hypotheses

We considered both hypotheses related to the change between week 1 and week 10 and hypotheses related to the comparison of the three groups. The primary hypotheses that we aimed to confirm were: 1) The score will change between week 1 and week 10 in the control group. 2) The score will not substantially change between week 1 and week 10 in the SOP-group and the SW-group, respectively, formalized as equivalence testing, where a change of less than 1 point was considered ignorable. The secondary hypotheses to be confirmed referring to the comparisons between the three groups were: 1) The change between week 1 and week 10 will not be substantially different for the SOP- and SW-groups (formalized again as equivalence testing, again with a difference of 1 point being considered ignorable). 2) The change between week 1 and week 10 will be different for the SOP- and SW-group, respectively, and the control group.

### Sample size

For sample size calculation it was assumed that all within-group average scores are normally distributed [21]. Further, it was assumed that the standard deviation of the change equals approximately 1 within all three groups. A difference of 1 was considered relevant for testing of the hypotheses. For sample size calculation in the context of equivalence testing, differences of zero were assumed (perfect equivalence), and the significance level was fixed to 0.025 corresponding to the recommended use of 95% confidence intervals in the equivalence testing procedure. For all other tests the significance level was set to 0.05. To reach a power of 80% a group size of *n* = 22 is required for both SOP and SW groups, while *n* = 17 are needed for the control group. The group sizes were thus chosen as *n* = 18 (control group) and *n* = 24 (SOP and SW groups) to account for 5% dropout.

### Statistical analysis

All analyses were performed with R (version 4.0.3). All statistical tests were performed based on 95%-confidence intervals (CI) for the mean changes between scores at week 1 and week 10 or for the between-group differences of mean changes. CIs were derived assuming normality of the endpoints. The null hypothesis was rejected if 0 was not in the CI (for equivalence testing: if the CI was included in [-1;+1], following the standard procedure for equivalence testing). Additionally, the average and standard deviation were computed in the different groups. Cohen’s *d* was calculated for effect size when t-tests were performed.

## Results

**Fig. 2:**
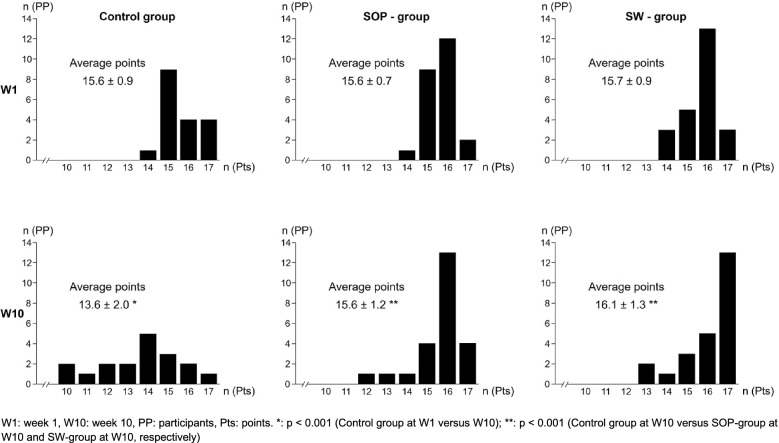
OSCE 1 and OSCE 2 results at week 1 and week 10 of the control group, the SOP-group, and the SW-group

Of the 66 participants, almost two thirds were female (*n* = 42). The ratio of women to men within the groups was: 11 to 7 (control group), 15 to 9 (SOP-group), and 16 to 8 (SW-group). The distribution of the achieved point scores of the three groups in the two OSCEs (week 1 and week 10) is shown in the form of histograms (Fig. 2). The control group reached a significantly lower (*p* < 0.001) score in OSCE 2 (week 10, 13.6 ± 2.0 points) than in OSCE 1 (week 1, 15.6 ± 0.9 points), resulting in an average difference of -2.0 (CI: [-2.9;-1.1]). This effect was very large (Cohen’s *d* = 1.3). The SOP-group reached the same average score in OSCE 2 (week 10, 15.6 ± 1.2 points) and OSCE 1 (week 1, 15.6 ± 0.7 points), i.e. the average difference was 0 (CI: [-0.63;+0.63]). For the SW-group, the average difference between the score in OSCE 2 (week 10, 16.1 ± 1.3 points) and OSCE 1 (week 1, 15.7 ± 0.9 points) was + 0.4 (CI: [-0.29;+1.12]), thus confirming that the score did not substantially decrease between week 1 and week 10.

Compared to the control group, the change of the score was significantly (*p* < 0.001) different in the SOP-group (average difference: +2.0, CI: [+0.9;+3.1]) and the SW-group (average difference + 2.4, CI: [+1.3;+3.6]), thus confirming the utility of both, the SOP and the second workshop for skills retention. Both differences had a very large effect (Cohen’s *d* = 1.2 and Cohen’s *d* = 1.3, respectively). The difference between the SW-group and the SOP-group (average + 0.4, CI: [-0.5;+1.3]) in OSCE 2 (week 10) was not significant, but the CI was not included in [-1;+1]. Therefore, a superiority of the second workshop over reading an SOP could neither be established nor excluded based on the available data.

## Discussion

Our study showed that a workshop on skills that need to be mastered for guideline-based therapy of cardiac arrhythmias yielded excellent results. When the participating interns were evaluated with an OSCE testing SC and TCP after the workshop, they reached a score of approximately 91.7% (15.7 points of a maximum of 17 points). It has been described that the management of tachyarrhythmias can be learned quickly with skills training [22]. The treatment of critical bradycardia with external pacemaker therapy can be safely taught by skills training with the manikin we used in our study [23, 24]. However, skills regarding resuscitation in general, basic live support of adults and children, basic neurological assessment, and the placing of intravenous lines are lost after a relatively short period of time [7, 9, 11–13, 15, 25, 26]. We also observed a significant loss of SP and TCP skills 10 weeks after our workshop in interns who did not receive an additional intervention, when performance of these skills was assessed in a second OSCE.

To maintain acquired skills, several methods have been described. Most of them are time and personnel-consuming. The best-studied method for skills retention is to repeat the skills training [12, 27–29]. Other means for successful skills retention include watching training videos, e.g., for cardiopulmonary resuscitation (CPR) [13], or watching a video of one’s own CPR performance during a previous training [14]. Repeated assessments of CPR skills, e.g., in an OSCE, also increased retention [30], presumably because of the testing effect [31]. Feedback during CPR trainings also led to longer skills retention, i.e., 1 year [15], while feedback during laparoscopy training only increased participants’ immediate performance but had no long-term effect [32]. Our study showed for the first time that reading an SOP had approximately the same impact on preventing skill loss than repeating the skills training of SP and TCP before a second assessment.

Since in our study reading an SOP on SP and TCP led to nearly the same maintenance of skills in interns 10 weeks after the initial workshop as repeating the workshop one week before the second assessment, reading an SOP provides an effective and inexpensive way for the retention of these skills. The costs for one workshop to train six participants amount to approximately 400 euros, while SOPs are usually part of a hospital’s quality management system [33, 34]. SOPs found their entry in the medical field to create standardized treatment strategies [17] and could, according to our data, also be used as teaching tools for skills retention. Since SOPs have been shown to improve the compliance to follow treatment protocols [35], undergraduate medical students could additionally benefit for their learning from getting accustomed to reading SOPs for skills retention. Furthermore, reading an SOP could easily be combined with other proven methods for skills retention, e.g., watching training videos or receiving feedback during a workshop [13, 15].

Our study has several limitations. Student participation was voluntary and could have led to inclusion of very motivated and skilled participants. We were not able to carry out a pre-test of our assessment because SC and TCP are not taught in the regular undergraduate curriculum at our medical school. This could be circumvented in future studies by carrying out a pre-test with physicians who perform these skills regularly. For the second OSCE (week 10), we had to use three different examiners for organizational reasons like in regular OSCEs. This could have led to some variability in the results despite standardized instructions how to use the checklist, but did not, which underscores the quality of the checklist. As it is usual for OSCEs to have different examiners, high quality checklists are the most important feature for OSCE scoring and rater training is a requirement for quality control [36]. Another option would be to have the same rater who assesses all participants, which is much harder to achieve. A strength of our study is that a high degree of standardization was maintained throughout the study. All participants were trained for SC and TCP and tested (OSCE 1) by the same physician. The OSCE checklist was standardized and validated with the available literature [5, 24, 37]. The three examiners of OSCE 2 were blinded to group membership of the participants. The participants were divided into the three experimental groups by stratified randomization based on their OSCE 1 results. Furthermore, the ratio of female and male participants was similar in the three groups. Before the study, a power analysis was carried out to determine the required number of study participants per group, which was reached for all groups and strengthens our findings. Further studies are needed to test whether reading an SOP is effective for long term retention of other complex skills compared to repeated training as the current gold standard for many skills. How frequently an SOP should be re-read for appropriate long-term performance of a complex skill will also have to be explored by additional studies.

## Conclusions

Synchronized cardioversion (SC) and transcutaneous pacing (TCP) skills were sufficiently acquired by final-year medical students in a workshop. A loss of these skills could be completely prevented by either a repeated training or by reading an SOP. In this context, the SOP is a cost-effective and resource-friendly method for skills retention. Whether reading an SOP is effective for retention of other complex skills needs to be investigated in future studies.

## Data Availability

All data generated or analyzed during this study are included in this published article and its supplementary information files.

## Abbreviations

ALS: Advanced life support
BLS: Basic life support
CI: Confidence intervals
CPR: Cardiopulmonary resuscitation
OSCE: Objective structured clinical examination
SC: Synchronized cardioversion
SOP: Standard operating procedure
SW: Second workshop
TCP: Transcutaneous cardiac pacing
